# Polymeric Thin Films for Organic Electronics: Properties and Adaptive Structures

**DOI:** 10.3390/ma6031159

**Published:** 2013-03-22

**Authors:** Sebastiano Cataldo, Bruno Pignataro

**Affiliations:** Department of Physics and Chemistry, University of Palermo, V.le delle Scienze, Bld. 17, 90128 Palermo, Italy; E-Mail: sebastiano.cataldo@unipa.it

**Keywords:** morphology, transistors, solar cells, thin films, solution processes, plastic electronics

## Abstract

This review deals with the correlation between morphology, structure and performance of organic electronic devices including thin film transistors and solar cells. In particular, we report on solution processed devices going into the role of the 3D supramolecular organization in determining their electronic properties. A selection of case studies from recent literature are reviewed, relying on solution methods for organic thin-film deposition which allow fine control of the supramolecular aggregation of polymers confined at surfaces in nanoscopic layers. A special focus is given to issues exploiting morphological structures stemming from the intrinsic polymeric dynamic adaptation under non-equilibrium conditions.

## 1. Introduction

The discovery in 1976 by Heeger, MacDiarmid and Shirakawa [1,2] of conductive polymers opened a new field of research at the boundary between chemistry and the physics of condensed matter. These materials display the electrical and optical properties of metals or semiconductors and preserve the desirable mechanical and processing properties of polymers. The growing interest in this class of materials has led to the origin of a new technology as well as a new market, namely plastic electronics [3].

From a structural point of view, these materials are generally characterized by a carbon-rich backbone which offers mechanical flexibility and by a π-conjugated electronic system that is able to access the full range of electrical properties, from insulators to metals, depending on their molecular structure and/or doping condition [4]. Indeed, the possibility to tune the molecular structure to obtain the desired electro-optical properties, the mechanical flexibility along with the wide availability and solution processability of these materials enables the realization of a wide landscape of low-cost, lightweight, flexible and even disposable organic devices such as LEDs [5,6], displays [7,8], bio-/sensors [9,10], transistors [11] and solar cells [12].

Essentially, the beating heart of all these devices consists of thin-films (about 100–300 nm in thickness), deposited on suitable substrates (e.g., glass or plastics) and composed of either a single or a blend of two or more polymeric materials depending on the type of device and application. However, it is well known that the properties of a thin film do not depend only on the physico-chemical properties of the constituent materials but also strongly on the film nanostructure needing to be optimized. Thus, the developing of organic electronic devices must directly involve the nano-engineering of thin films [13,14]. For this reason and also pushed by the requirements of an emerging new market, scientific and technological effort has became increasingly more intense in the set-up of new strategies not [15,16].only able to control the morphology but also enabling process scale-up for large scale production

The structural assembly of a thin-film involves a complex system and the control of its molecular order requires a supramolecular approach [17]. At first, in order to introduce inside the thin-film the required structure-related functions, it is necessary to act on both the intermolecular and molecule/substrate interactions [18,19]. Then, by exploiting the feasibility of solution-processing across a bottom-up approach, two main self-processes can be exploited, namely *thermodynamic* self-assembly (steady-state structures) and *dynamic* self-organization (non-equilibrium structures) [20]. Importantly, while the first is limited to those molecular arrangements close to the energy minimum, dynamic self-organization may lead to the development of a large spectrum of non-equilibrium thin-film structures. Thermodynamic self-processes are quite easy to develop and lead to a relatively simple organization, whereas the non-equilibrium ones are characterized by structural fluctuations allowing even ordered geometries like periodic super-structures or supra-molecular aggregates [21]. Thus, by freezing these complex structures on a given substrate, it is possible to make an *artificial selection* among infinite energy levels (in principle) corresponding to as many thin-film organization paths [22].

Recently, literature has developed a new lexicon to indicate new classes of self-assembly phenomena. Thus for instance, the expression “constitutional dynamic chemistry” refers to systems able to react to external stimuli by modifying their structure through the exchange or reorganization of components and leading to different final configurations [23]. Another example is Directed Self-Assembly (DSA) which consists of accurately driving self-organization through the careful design of the molecular structure of building blocks [24]. Also, through Controlled Evaporative Self-Assembly (CESA) it is possible to arrange ordered structure by controlling the solvent evaporation kinetics of solutions confined at surfaces [25]. However, these represent only some examples, since such a vocabulary is continuously increasing as an indication of the research effort in this field.

By the comprehensive expression *artificial selection* cited above, it is intended that the opportunity to program the nanostructural order is given by driving the assembling system along one of the possible configurations eventually toward a far-from-equilibrium state. A structure can be frozen in a far-from-equilibrium state exploiting speed as a threshold parameter in developing different aggregation pathways using a proper experimental protocol. Thus an ordered structure may be frozen on a surface by acting on the kinetic parameters of the deposition process (as solvent evaporation rate, film-transfer speed *etc*.) which in turn bias the competing forces involved in the structural evolution (intermolecular and surface-molecule electromagnetic forces, chemical potential gradients *etc*.) [26]. These competing forces arise from the chemical nature of the building-blocks (*chemical pressure*) and from the physical parameters of the process (*physical pressure*), that have to be conveniently balanced to achieve the desired nanostructures. Then, by experimentally exploring the effect of changing physical and/or chemical parameters it is possible to find, for a given system, a multitude of structural shapes and domain sizes [14,27,28].

Here, we deal with two major players of organic and plastic electronics, namely organic thin film field-effect transistors (OFETs) and organic solar cells (OSCs).

In particular, OFETs are becoming increasingly important for the development of low-cost new applications as flexible displays [7,8] sensors and bio-sensors [9,10] even with a disposable view.

As for OSCs, their potential contribution to the world energy issue and features such as cost-effectiveness and easiness in large-area fabrication, make them imaginable as future renewable energy sources, especially for the developing countries [29,30]. Furthermore, many original applications as building-integrated photovoltaics, stand-alone power sources for portable devices or remote applications can be envisaged thanks to interesting features like semi-transparency, lightness, thinness, flexibility and eco-compatibility [31,32].

This review focuses on the role of the thin-film morphology in OFETs and OSCs. It aims to show the relation between molecular 3D order and device performance, reporting some significant case studies from the most recent literature, which encompasses some of the principal solution methods for organic thin-film deposition and structural manipulation.

## 2. Morphology and Device Performance: Relations and Optimization

### 2.1. Organic Field Effect Transistors 

In 1947, John Bardeen, William Shockley, and Walter Brattain invented the transistor bringing to history one of the major discoveries of the last century. Actually, their invention marked the birth of modern electronics, the transistor being its principal component. In the last sixty years, research and microelectronic manufacturing has briskly developed, passing from the first centimetre-sized Ge-based device to microprocessors containing hundreds of millions of transistors. Inorganic electronics, however, is characterized by some technological limits (costs, weight, stiffness) while the employment of organic semiconductors in FETs would bring different advantages. In order to enhance the performance of OFETs, it is essential to maximize the charge carrier mobility by inducing order in the organic semiconductor. However, while it is possible to obtain single crystals of organic small molecules [33,34,35,36], the achievement of long-range order in polymers is a difficult task since they generally show microcrystalline domains embedded in an amorphous matrix [37]. This matrix hinders charge transport between neighbouring domains leading to low mobility. Still, polymers afford very important advantages as polymer-based OFETs can be easily processed at low temperature from solution. All this is very desirable for plastic electronic applications including systems like flexible displays and electronic papers [38,39,40]. Furthermore, the possibilities given by organic synthesis to choose the material properties “*à la carte*”, permits materials to be wisely tailored for specific challenges.

There are a large number of factors that influence the performance of an OFET. For example, the choice of metal electrodes for efficient charge injection [41], the dielectric material influencing field-effect properties and charge trapping [42,43,44], and most importantly the choice of organic semiconductor which plays a key role in transistor design and performance. Actually, the organic semiconductor charge carrier mobility (μ) crucially determines the OFET performance including current modulation and switching speed. While the charge transport in inorganic semiconductors is generally agreed to occur via delocalized electronic bands, limited by lattice defects and vibrations, the charge mobility in organic semiconductors is usually triggered by π-conjugated molecular orbitals affording the right percolation pathways between source and drain electrodes. Obviously, charge mobility is likely high when charges move between orbitals without sensible hindrance, that is usually by providing the highest intermolecular overlap between π-orbitals, thus mimicking the typical band charge transport of inorganic semiconductors. Unluckily, intermolecular van der Waals forces in organic semiconductors are much weaker than covalent bonds in the inorganic ones (*i.e.*, silicon or germanium) and thermal fluctuations (electron-phonon coupling) are able to avoid molecular order and π-π coupling (especially for weakly interacting molecules) causing lower mobility than that observed in inorganic crystalline semiconductors. By failing to have an effective π-π coupling, charges would travel between molecules through a slow phonon-assisted hopping mechanism [45,46,47]. In this view, the optimization of orbital overlap, responsible for an effective charge transport, would maximize charge mobility.

**Figure 1:**
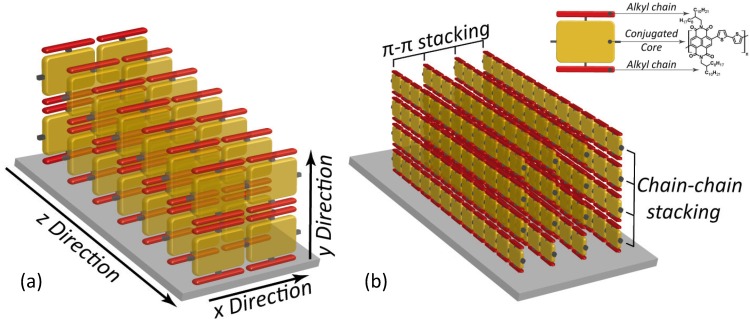
(**a**) Schematic depiction of charge transport in a edge-on packed polymer assembly. Yellow bricks indicate monomers along the polymer backbone. X, Y, Z denote the charge transport directions along the polymer chain, across the alkyl moieties of parallel chains and between face-to-face piled chains, respectively; (**b**) Schematic depiction of the π-stacking and chain-to-chain packing of polymers. In the inset, the reading-key shows the example of P(NDI2OD-T2) that is discussed below.

In order to understand some more details of charge transport mechanisms in polymeric thin films, let us refer to the peculiar well-ordered configuration depicted in Figure 1, showing polymer chains packed face-to-face in an edge-on structure with respect to the dielectric substrate. As indicated by the red arrows, for such a structure charge carriers may move along three different directions in response to an electric field:

(i) Intrachain transport along the molecular backbone (x-direction in Figure 1a). Here, in an ideal chain the whole molecule is wrapped by a unique conjugated π-orbital allowing fast charge diffusion. Unluckily, molecular defects like twisted bonds or steric effects may affect π-conjugation, thus cutting down charge mobility. For this reason, a polymer chain may be conveniently divided into different conjugated fragments, whose extension would represent the conjugation length, the intrachain charge current taking place by hopping between neighbouring conjugated sections. Therefore, intrachain transport is a major component of charge mobility and quality and extensive chain alignment may provide higher conjugation length, then fast charge motion; (ii) Interchain transport along the π-stacking direction (z-direction in Figure 1a) is also important for charge mobility, although a slower rate is expected in this way. Here, face-to-face stacking and interchain distance are crucial. Indeed, a narrow separation between parallel chains leads to an extended π-π stacking and thus to a high mobility. With this view, strictly-packed self-assembled polymers may allow high performance FETs; (iii) Finally, the structure depicted in Figure 1a agrees with the picture in which charge transport along the y-direction brings only a small contribute to the whole charge mobility. High performance semiconducting polymers are often functionalized with lateral alkyl chains in order to improve solubility, and these solubilizing groups are usually electrical insulators hindering the diffusion of charge carriers across the chain-to-chain direction. In addition, this insulator effect increases with the steric hindrance of the substituents. Therefore, since the transport mechanism is mainly controlled by the interchain transport in the π-stacking direction and the intrachain trasport along the molecule backbone, it is expected that the optimization of these two features would lead to high charge mobility values.

Depending on the assembly optimization, charge mobility can span over several orders of magnitude according to the materials used for the semiconducting channel [48]. Indeed, it can range from 10^−4^ cm^2^/(V s) or lower for weakly conductive organic semiconductors to the range ~1–10 cm^2^/(V s) for the well-ordered ones [49].

What the above reveals is that despite the important role played by other factors such as contact resistance [50], interface charge trapping [51,52] and dielectric properties [43,44,53,54,55], supermolecular structure is fundamental [56]. Essentially, the structure control depends not only on an accurate chemical design which looks at the specific intermolecular interactions to achieve an effectively ordered polymer self-assembly, but also on the right deposition method. Indeed, by suitable thin-film processing, molecules can be driven to long-range organization. Moreover, regardless of the method employed to deposit the organic material, the structure can also be improved after deposition. In this regard, thermal annealing [57,58] is frequently used just to exploit the molecular reorganization induced by thermal motion. Polymer semiconductors are typically annealed around glass transition/melting temperatures. Furthermore, solvent-annealing has also been proved to enhance the structural properties, and then to improve the device performance [59,60].

In this way the structure can be controlled by reducing amorphous boundaries by pursuing the principle that the stronger/extended the intra- and inter-chain π-π coupling, the higher is the charge mobility.

In addition it has to be noted, although to a lesser extent, that the structural nature of the dielectric layer may also be important for the OFET’s performance. Thus, the dielectric chemistry, roughness, morphology, polarity along with other parameters may affect the overall self-structuring of the organic thin films [61,62]. Also, effort on dielectric systems is being continuously spent to develop new high-k gate insulators, which in turn can be very useful in developing low voltage transistors or other devices [43]. In any case, usually gate insulators are functionalized by self-assembled monolayers or other surface agents able to lower the dielectric surface tension thus minimizing the interaction with the active semiconductor along with the charge trapping. Nevertheless, an exhaustive discussion on this point goes beyond the scope of this review, which is intended to focus on how deposition methods may affect the structure and function of the active semiconducting thin film.

Although a lot of experimental and theoretical studies have been accomplished, the correlation between supramolecular interactions, film morphology and the comprehension of charge transport mechanism still remains a complex matter and, in some experiments, contrary to what might have been expected, poorly ordered films have shown higher performance.

**Figure 2:**
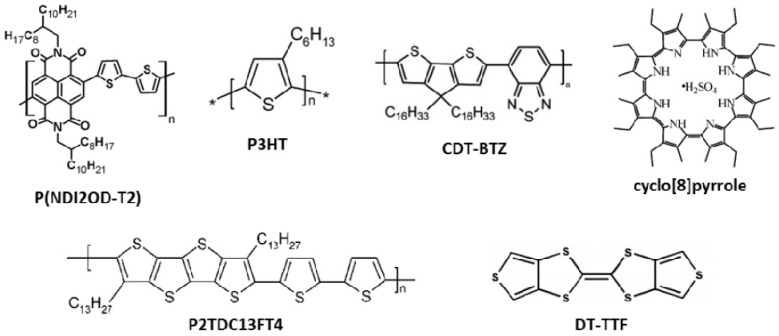
Molecular structures of materials used in organic thin film field-effect transistors (OFETs) reported as examples in this review.

**Figure 3:**
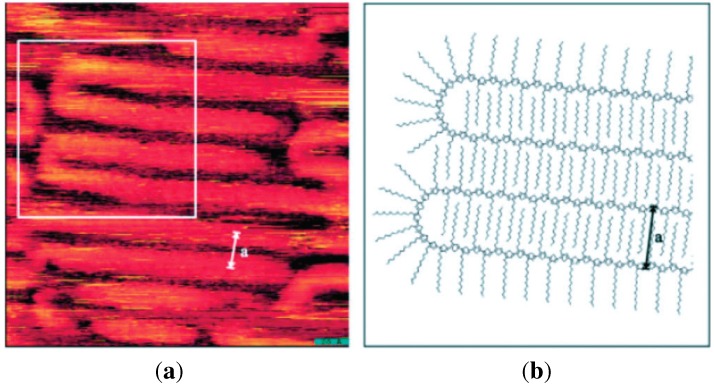
(**a**) Scanning tunneling microscope image of chain folding for regioregular poly(3-dodecylthiophene); (**b**) Calculated model of chain folding (Reprinted with permission from reference [64]. Copyright 2002 Wiley-VCH Verlag GmbH & Co.).

Self-assembling in polycrystalline lamella architectures might be expected to be the optimal packing for an efficient charge transport. Up to date, among the most investigated semiconductor polymers is the regioregular poly(3-hexyl-thyophene) (P3HT; Figure 2). It is also one of the reference materials for the OFET market, thanks to its high solubility and performance. P3HT showed one of the highest charge carrier mobilities recorded for a polymer OFET, with values of about 0.1–0.2 cm^2^/(V s). Sirringhaus *et al.* [63] first demonstrated these values, while also comparing thin films deposited by methods allowing a different kinetic control on the molecular self-assembly of the polymeric layer. P3HT has been deposited through both spin-coating, which typically froze the system toward a dynamic adaptive one, and solution-casting which has a lower kinetic and allows the system to self-organize, in principle, closer to its energy minimum. Actually, the self-organization of the polymer going from solution into the solid state was found to be greatly dependent on the deposition process, which led to different order and degree of crystallinity. Authors found that solution-casting affords both higher crystallinity and charge mobility than spin-coating. The high charge mobility achieved, a worthy 0.1 cm^2^/(V s), was essentially attributed to the head-to-tail regioregular conformation of the P3HT which likely self-assembles in a 2D thermodynamically favoured lamellar structure (see Figure 3) [64,65].

**Figure 4:**
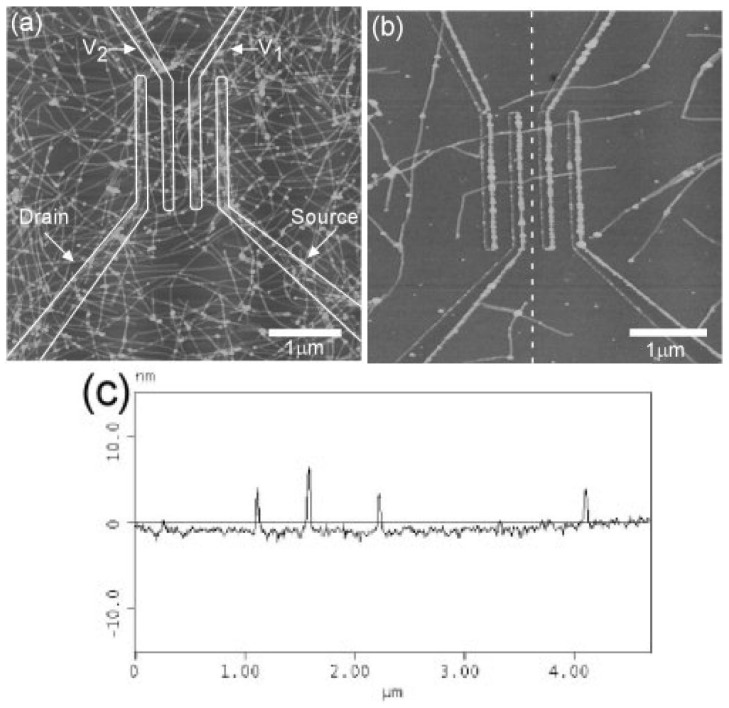
(**a,b**) AFM image of P3HT nanofibers across electrodes on SiO_2_/Si substrate; (**c**) cross section of the dashed line shown in (b). The fibre heights from top to bottom are 5.6, 6.3, 3.9, and 3.3 nm. (Reprinted with permission from Reference [67]. Copyright (2003) by the Wiley-VCH Verlag GmbH & Co.).

Thus, authors demonstrated that better control of such a structural anisotropy, allowing for a truly delocalized transport, would be the route to high mobility. Still, regioregular P3HT has shown even higher charge mobility. For instance, Wang *et al.* [66] obtained a hole mobility of about 0.2 cm^2^/(V s) for 2–4 nm thin-films with increased on/off ratio after thermal treatment, depositing P3HT by dip-coating. Such a procedure allowed an almost stable crystalline structure (presumably close to equilibrium) of the thin film at the interface with the SiO_2_ gate insulator with an improved structural order. Furthermore, depending on the solvent, P3HT has shown the formation of fibrillar structures (see Figure 4), which have also been employed in FET. Nanofibers of regioregular P3HT were deposited onto SiO_2_/Si substrates by casting from dilute *p*-xylene solutions [67] yielding a fibre network displaying hole mobility of about 0.06 cm^2^/Vs with on/off current ratios greater than 10^3^. Besides P3HT, other conjugated polymers show similar fibre structures which are believed to be induced by π-stacking supramolecular interactions [68].

**Figure 5:**
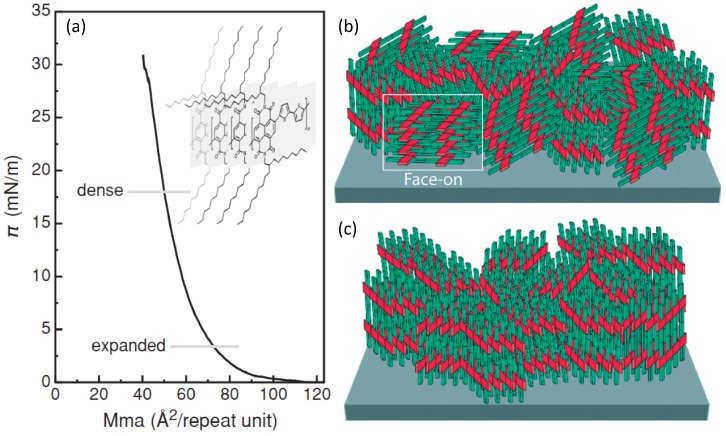
(**a**) Superficial pressure *vs.* mean molecular area graph showing the rise in pressure as the polymer self-assembly proceeds toward the edge-on packing. (Reprinted with permission from Reference [69]. Copyright (2012) by the Wiley-VCH Verlag GmbH & Co.); (**b,c**) Descriptive depictions respectively of a mixed face-on/edge-on structure and of a strictly packed edge-on structure (Reprinted with permission from Reference [70]. Copyright (2006) by the Nature Publishing Group).

Actually, in the last few years, higher mobility values have also been achieved by employing n-type polymers. Fabiano *et al.* [69] exploited controlled deposition methods to build monolayer and multilayer field-effect transistors using an air-stable, soluble n-type polynaphtalene-bithiophene (P(NDI2OD-T2); N2200 ink by Polyera; Figure 2). By using Langmuir Schaefer (LS), a monolayer FET with a vertical (edge-on) alignment of the P(NDI2OD-T2) molecules with respect to the dielectric surface underneath (Figure 5a,c) was experienced.

**Figure 6:**
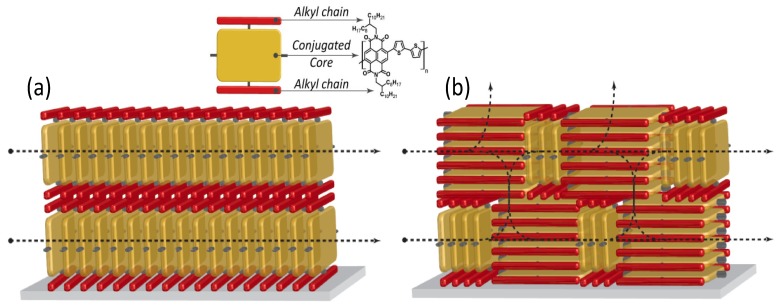
Graphical depiction showing the charge carrier paths available in (**a**) an edge-on; and in (**b**) a mixed edge-on/face-on ordered thin-film (tilted phases are missing in this scheme for simplicity). In the inset, the reading key is reported by using again the example of P(NDI2OD-T2).

Indeed, the surface pressure exerted by two Teflon barriers at the air water interface on the polymer chains, allows accurate control of the long-range molecular order and then the self-assembly of densely packed monomolecular films with predominant edge-on alignment over large areas. The obtained edge-on packing was proved to be quite stable indicating the achievement of a close to thermodynamic minimum-energy state. In fact, the thermal annealing treatment had minimal effect on the film morphology and surface roughness. The authors showed monolayer FETs exhibiting good injection properties, relevant current on/off ratio >10^3^ and a remarkable long lasting (five weeks) in-plane saturated electron mobility of about 3 × 10^−3^ cm^2^/(V s) in a monomolecular layer (about 3 nm thick, *i.e.*, the molecular lateral size) FET. By increasing the number of layers up to 15, carrier mobility was observed to grow up to a plateau starting from six layers at 2 × 10^−2^ cm^2^/(V s), the switch-on voltage of the field-effect changed from +28 V (monolayer) to +16 V (15-layers FET) approaching that of spin-coated P(NDI2OD-T2) transistors. Although a significant value, the 15 layers mobility achieved in the LS edge-on configuration, is about one order of magnitude lower than that commonly observed for far-from-equilibrium thin film structure, prepared by spin-coating with the same P(NDI2OD-T2) polymer. Actually, typical charge mobility of such a device, which shows also weak ambipolar behaviour [71] is about 0.45–0.85 cm^2^/(V s). Through a dynamic self-organization process, spin-coating yields a dominant face-on (parallel to the surface, Figure 5b) orientation of the P(NDI2OD-T2) chains. This has been demonstrated again by a thermal annealing experiment, beyond the melting point of P(NDI2OD-T2), which led in fact to a shift from largely face-on packing to a pronounced edge-on texture [72]. Furthermore, LUMO levels, rising due to the edge-on conformation, may also negatively affect charge injection [73]. Although the edge-on molecular packing was found to be beneficial for several high performance polymer FETs due to the fast two-dimensional charge transport along the chain backbone and the π-stacking direction, Fabiano *et al.* [69] showed that such ordering for P(NDI2OD-T2) may be less efficient for transport in multi-layered (about 10 layers) structures whereas it is a fundamental for monomolecular layer FETs. Actually, the anisotropy of P(NDI2OD-T2) in multilayered LS devices limits the charge transport to the in-plane direction (Figure 6a) with inefficient out-of-plane transport between backbones separated by the insulating long octyl-decyl side chains (red bars in Figure 6) [74].

Better, a non-equilibrium face-on rich arrangement (spin-coated films) leads to three-dimensional charge transport through adjacent layers that are coupled by the out-of-plane π-stacking (Figure 6b) [75]. This directly entails an enhanced in-plane and out-of-plane electron mobility, as confirmed by bulk charge transport measurements [76]. Thus, the lower electron mobility for LS multilayer FETs would depend on the reduced interchain vertical transport that lowers the number of efficient percolation paths.

**Figure 7:**
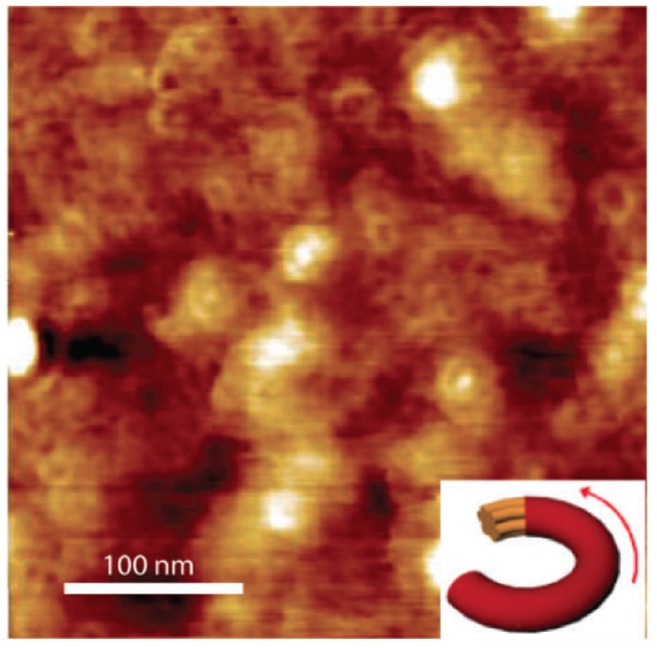
AFM image of spin-coated CDT–BTZ–C16 copolymer film showing ring-shaped structures (Reprinted with permission from Reference [78]. Copyright (2012) by Wiley-VCH Verlag GmbH & Co.).

**Figure 8:**
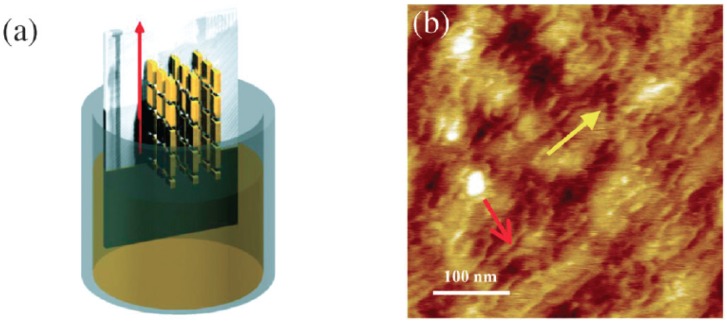
(**a**) Graphical depiction of the CDT-BTZ deposition by dip-coating showing the polymer backbones aligned parallel to the dipping direction; (**b**) AFM image showing the polymeric fibres (red arrow) aligned along the dipping direction (yellow arrow) (Reprinted with permission from reference [78]. Copyright (2012) by Wiley-VCH Verlag GmbH & Co.).

As for other examples comparing well-defined structures in different kinetic configurations giving significant charge mobility, we can report the case of poly(2,5-bis(thiophene-2-yl)-3,7-ditridecanyltetrathienoacene (P2TDC13FT4; Figure 2) [77] and cyclo-penta-di-thiophene-benzothiadiazole copolymer (CDT-BTZ; Figure 2) [78]. P2TDC13FT4 spin-coated thin-films show a mobility exceeding 0.3 cm^2^/(V s). As for CDT-BTZ, spin-coating deposition allows dynamic organization of molecules in ring-shaped structures (see Figure 7) whose film presents a mobility as high as 0.67 cm^2^/(V s), probably because of the reduced number of trapping and scattering sites. Nevertheless, the same CDT-BTZ is observed giving even higher mobility if deposited by dip-coating (Figure 8a) [78]. Different from the spin-coating deposition, dip-coating is a slower film-forming process. Moreover, the choice of a solvent with a suitable boiling point allows kinetic control of film formation, in which the structure depends on the solvent evaporation rate as well as on the dipping speed. In this way, dip-coated CDT-BTZ showed fibre-like structures aligned parallel to the dipping direction (Figure 8b) (instead of the ring structure of the spin-coated film), constituted by a lamellar edge-on packed polymer. The reduced grain boundaries, the enhanced crystalline order and the anisotropy of elongated fibres lead to a charge mobility as high as 1.4 cm^2^/(V s) along the alignment direction, which is more than twice the value reached for the ring-shaped structures by the spin-coating process.

**Figure 9:**
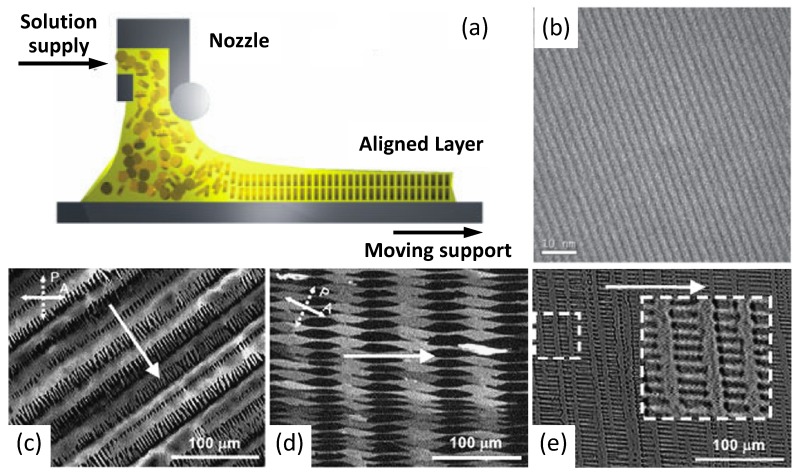
(**a**) Schematic representation of the zone-casting deposition method; (**b**) TEM large-area microgragh of a linear pattern. (Reprinted with permission from Reference [79]. Copyright (2005) Wiley-VCH Verlag GmbH & Co.). (**c,d,e**) Optical micrographs of microcrystalline columnar patterns of DT-TTF deposited on glass by zone casting at different casting rates: (**c**) 6 µm/s; (**d**) 10 µm/s; (**e**) 16 µm/s. Arrows indicates the casting direction (Reprinted with permission from Reference [80]. Copyright (2008) by Elsevier).

Nonetheless, dip-coating turns out to be unsuitable for poorly soluble high molecular weight polymers because of the need for high solvent temperatures avoiding an evaporation rate control. In this case, controlled film formation has been carried out by zone-casting deposition [79] (Figure 9), which permits control of both the temperature of solvent and substrate. As evidenced in the drawing of Figure 9a, a nozzle can move along the substrate surface dispensing the polymer solution. Furthermore, tuning parameters such as the rate of nozzle refuelling and the speed of the underlying substrate enable gain of precise control of the film deposition as well as obtaining the formation of complex patterns (Figure 9b–e). Among other methods, zone casting has been exploited to build OFETs based on dithiophene-tetrathiafulvalene (DT-TTF; Figure 2) [80] and pentacene, obtaining long-range ordered features showing mobilities respectively of 0.17 and 0.4–0.7 cm^2^/(V s) with a noticeable on/off ratio of 10^6^–10^7^ in this latter case.

In order to achieve a thin film structural and morphological control, Langmuir-Blodgett (LB) deposition is one of the finest methods allowing the deposition of molecular thin-films in a close-to-equilibrium state, and it has been employed in the preparation of several devices [81,82,83,84,85]. Moreover, it is well known that the employed solvent may importantly affect the final film structure. For example, OFETs based on ultrathin-films consisting of multilayered P3HT with the typical lamellar structure were realized, comparing different solvents, by Xu *et al.* [83] reaching a maximum mobility of 0.02 cm^2^/(V s) with xylene as solvent. Nevertheless, despite their potential in structure control, semiconductor polymers have proved to be unsuitable for LB deposition. Much better results have been obtained with this technique for organic small molecule FETs. Indeed, LB films of cyclo[8]pyrrole [81] (Figure 2), which showed an ordered layer structure parallel to the substrate, gave an outstanding charge mobility of 0.68 cm^2^/(V s). Besides, OFETs have been developed using LB monolayer only 1.3 nm thick of copper phthalocyanine [82] showing a bulk-like mobility of 0.04 cm^2^/(V s) and a noticeable on/off ratio > 10^6^.

Whereas LB is a slow deposition process and unsuitable for large area deposition, this is not the case for inkjet-printing which is one of the most promising solution-based methods for the controlled deposition of organic semiconductors. Indeed, its capability for large scale production is very desirable for industry. Although inkjet-printing generally leads to amorphous structures, it has been demonstrated for pentacene FETs that it is possible to control the coverage uniformity of film morphology and the pentacene nanocrystal size by regulating the solvent evaporation rate; for example varying the temperature of the substrate, by adding suitable additives or by overlapping printed droplets [86,87]. In this way, a mobility enhancement up to threefold has been observed. However, in this respect it is important to note that in the case of polymers some poorly ordered thin films far-from-equilibrium have been reported to exhibit unexpectedly high OFET mobility. This is for instance the case of the above discussed spin-coated thin-films of the air-stable n-type polymer P(NDI2OD-T2) [71] showing ultimately a remarkable electron mobility of 0.85 cm^2^/(V s). Indeed, for this system, the wide-angle X-ray diffraction (XRD) scans revealed only negligible Bragg reflection intensities, indicating no long-range order and to explain charge transport in such systems different theoretical models have been proposed [88,89].

### 2.2. Organic Solar Cells

One of the major issues which the scientific community has been called to face this century, is to cope with growing worldwide energy consumption which, despite the economic crisis of recent years, is expected to increase at an annual rate of 1.5%–2.0%, on average [90]. Most of the energy used today, derives from the exploitation of non-renewable fossil fuels including oil, coal and natural gas which, at this rate will be fast exhausted. Moreover, and even more urgent, the combustion of fossil fuels for producing energy, pumps into the atmosphere a huge amount of greenhouse gases that are responsible for the global warming we are experiencing today (2–5 °C in this century). Conversely, solar light is green, renewable and with quite unlimited access. It is estimated that the sun pours onto the Earth’s surface an amount of energy so great that in one year it is about twice the energy obtainable from all the Earth’s non-renewable fossil resources [91,92].

Thus, one of the most promising strategies to tackle today’s energy issue would be the exploitation of solar energy by photovoltaic technology. Currently, the majority of photovoltaic cells are based on inorganic materials that, as in the case of transistors, have some limitations such as high material and manufacturing costs, which limit a broad diffusion of the technology (see for instance the Annual Energy Review by September 2012 from the U.S. Energy Information Administration) [93]. More recently, research has made an intensive effort towards the development of new low-cost PV technologies; organic solar cells (OSCs) are one of the most promising solutions.

**Figure 10:**
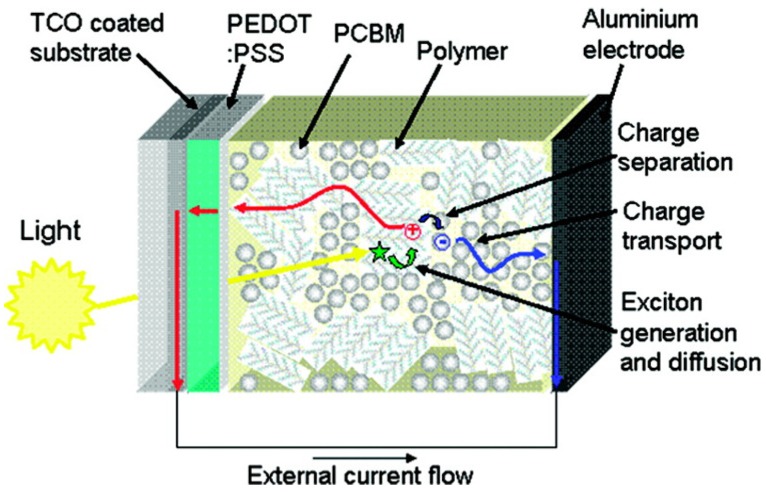
Schematic depiction of the photovoltaic operation for a bulk heterojunction solar cell. (Reprinted with permission from Reference [107]. Copyright (2008) by Materials Research Society).

As in the case of OFETs, OSCs are mainly based on semiconductor π-conjugated polymers, also coupled with other conjugated materials such as fullerene-derivatives or other organic conjugated molecules. In paragraph 2.1, we have already discussed the difference between organic and inorganic semiconductors in terms of charge transport mechanisms. Nevertheless, another major point concerns more precisely the photovoltaic application. In particular, organic semiconductors have low dielectric constants (ε ≈ 2–4), considerable electron-lattice interaction and electron correlation effects [94]. Therefore, while in inorganic materials the photoexcitation yields free charge carriers, in organic semiconductors it results in bound electron-hole pairs (Frenkel’s excitons) having a binding energy of about 0.3–1.0 eV [95,96]. This means that in the absence of a dissociation mechanism to produce free charges, most of the excitons would radiatively and/or non-radiatively recombine. This is the reason why the first primitive OSC, consisting of a single polymer layer and two electrodes arranged in a Schottky diode structure, had negligible power conversion efficiencies (PCE <<1%) [97,98]. This issue was partially fixed by Tang in 1986 [99], who introduced the concept of bilayer heterojunction. He obtained an appreciable efficiency of about 1% for a high-vacuum deposited Cu-Phtalocyanine/perylene-derivative electron-donor (D)/acceptor (A) bilayer device. Indeed, the difference between the donor ionization potential and acceptor electron affinity creates at the D/A interface a potential drop sufficient to split excitons. However, the efficiency of bilayer heterojunction OSCs is limited by the requirements of the exciton diffusion at the D/A interface. Suprisingly, organic semiconductors present an exciton diffusion length of about 10 nm meaning that after diffusing for such a distance, the exciton recombines. This limits the thickness and then the amount of light absorbed by the photoactive layer, since in a film thicker than the exciton diffusion length most of the excitons would not be able to reach the D/A interface. This drawback was fixed by Hiramoto [100] who introduced in 1992 the concept of bulk heterojunction (BHJ) by co-evaporating D and A materials in high-vacuum conditions. However, the first efficient BHJ OSCs were demonstrated in 1995 by Halls [101] and Yu [102] who respectively processed from solution a polymer:polymer and a polymer:fullerene D:A BHJ OSC. In the latter device, an impressive PCE of 2.9% was demonstrated along with the potential of fullerene-derivatives as A-materials. Today, solution deposited BHJs, using fullerene-derivatives as A component, dominate the scene and OSCs are now approaching remarkable PCEs of about 10% [103,104,105]. Very recently, Heliatek announced he had achieved a new world record of 10.7% [106]. The solution processability of these materials, which allows low-cost manufacturing and the possibility to use flexible and light substrates, makes these devices very attractive for the new market of plastic electronics [107,108]. Thanks to co-deposition by solution processes, the D/A interface of BHJs may be extended to the whole film, to a certain extent independently of its thickness, likely forming a bi-continuous interpenetrating network of D and A nanoscopic phases. This reduces significantly the distance that the exciton has to travel to reach the D/A interface, increasing the dissociation probability and thereby PCE. The conventional mechanism of photocurrent generation for a polymer:fullerene BHJ OSC is sketched in Figure 10. Photons are mainly absorbed by the D component to generate excitons diffusing across the film until the D/A interface where they may dissociate by transferring an electron to the fullerene-rich phase. Once generated, free holes and electrons migrate to the respective electrodes through the D and A phases generating an external current flow. Thus, from this Figure it is easy to understand that OSC PCE is affected by several factors including the D/A energy level offset, the materials absorption spectra, the total amount of light absorbed and the photoactive film thickness, but the role of the BHJ nanomorphology stemming from the material blending process is crucial for the achievement of high PCEs.

To date, a lot of research has been devoted to study the correlation between the thin film BHJ structure and the OSC’s performance [109,110], and it is widely accepted that control of the material phase separation is strategic to maximize the photocurrent. Essentially, the D/A interface extent and the material domain size must be balanced in order to reduce the exciton recombination favouring an effective charge photogeneration. Indeed, on the one hand large domain size (small D/A interface) would determine scarce exciton dissociation, since excitons may not reach a D/A interface due to their short diffusion length. On the other hand, a very thin phase separation (large D/A interface) may even increase the exciton recombination. In particular, if the domain size is smaller than the Coulomb capture radius, charges will not be able to escape their own attraction and may undergo recombination [111]. Afterwards, once free charges are engendered they need a continuous percolative path to the electrodes, otherwise electrons and holes would be trapped inside the film, recombining and thereby leading to current losses. Thus, in order to efficiently transport charges to electrodes, these paths would have to have the highest possible charge mobility. Concurrently, a proper balance of holes and electrons mobilities would help to suppress space–charge formation, drastically decreasing the PCE [112]. As a matter of fact, it has been recently demonstrated that in the case of all-polymer OSCs, a high fill factor may be achieved by using material blends with similar hole/electron mobilities [113]. Then, considering all these requirements, the ideal film morphology must be structured as a highly conductive, bi-continuous and interpenetrating network of D and A components with a phase segregation having a domain size on the same scale of the exciton diffusion length and with balanced hole/electron mobilities.

Although morphology control can be achieved by playing on many parameters, the optimization of such kind of architectures remains a challenging task. Even more so if we look at the growing evidence that a BHJ may be not the simple combination of two material pure phases with well-defined interfaces but rather a more complex system characterized by at least three phases, including the pure D and A as well as one or more amorphous intermixed ones [114,115,116].

The formation of a BHJ structure generally concerns a far-from-equilibrium organization of D and A systems and its optimisation passes through a fine balance between the thermodynamic and kinetic control of the thin-film formation.

Thermodynamic control may be achieved by designing suitable molecular structures (block copolymers, double-cable materials, *etc.*) [117,118,119,120] including functionalization to get improved D:A solubility and miscibility or by properly tuning the surface free energy of the substrate which may drive phase-separation. On the other side, kinetic control deals with the dynamic parameters employed during the thin film deposition. In addition to the instrumental speed parameters, these include important chemical properties like the solvent viscosity and boiling point, as well as the reorganization/crystallization in terms of diffusion rate of the components induced by post-deposition annealing procedures *etc*. In the following, some case studies are reported dealing with solution processed BHJ OSCs in order to illustrate the influence these factors may have on morphological control.

**Figure 11:**
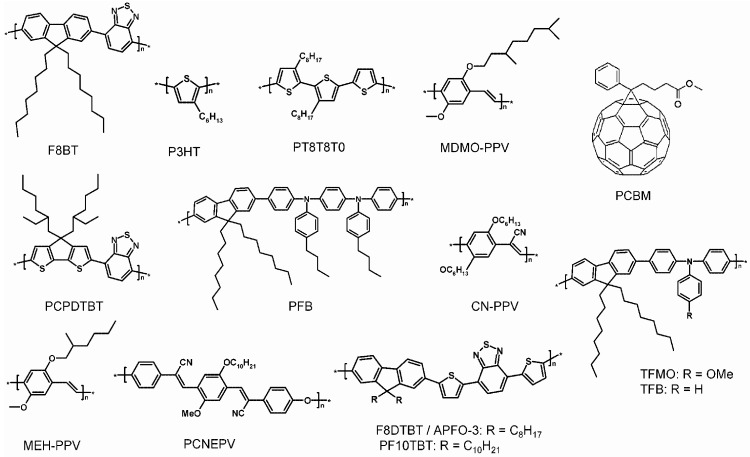
Some of the most used organic semiconductor materials in OSCs. (Reprinted with permission from Reference [110]. Copyright (2010) by American Chemical Society).

Until now, among the most widely studied systems, are those based on P3HT and poly[2-methoxy-5-(3’,7’-dimethyloctyloxy)-1,4-phenylenevinylene] (MDMO-PPV) as D and on the fullerene-derivative 1-(3-methoxycarbonyl)propyl-1-phenyl-[6,6]-methanofullerene (PCBM) as A components (Figure 11), which have shown among the highest PCE [121,122,123]. However, many other promising and innovative nano-materials (e.g., derivatives of carbon nanotubes [124], graphene [125,126] *etc*.) are potentially of emerging interest.

The effect of the solvent used for deposition has been widely demonstrated for MDMO-PPV:PCBM dyad, which showed a significant increase in PCE (from 0.9% to 2.5%) [121] by exchanging toluene with chlorobenzene.

**Figure 12:**
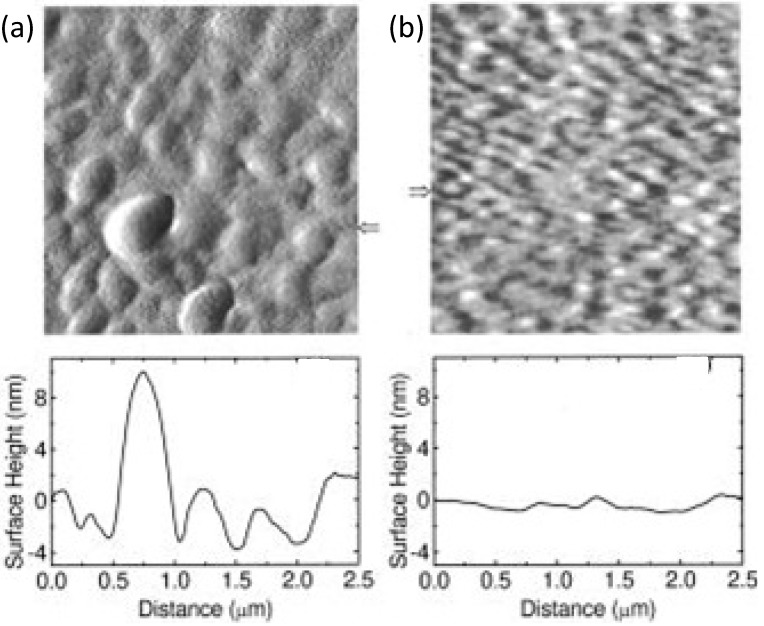
AFM surface morphology of MDMO-PPV/PCBM (1:4 wt%) BHJ thin-film spin coated with different solvents: (**a**) toluene; (**b**) chlorobenzene. The cross sections below the images are taken horizontally corresponding with the arrows. (Reprinted with permission from Reference [121]. Copyright (2001) by American Institute of Physics).

Actually when spin-coated from toluene, the BHJ shows a coarse morphology (see Figure 12a) since the well-known crystallization of PCBM hinders an effective exciton dissociation. Instead, by using chlorobenzene, a smaller and more favourable phase separation is achieved (Figure 12b) with a smaller PCBM domain immersed in a MDMO-PPV matrix; this has been attributed to the larger solubility of PCBM in chlorobenzene [121,127,128,129]. In addition, it has been considered that the solvent evaporation rate may drive the deposition speed which is a crucial factor for morphology. Indeed, a lower evaporation rate (longer deposition time) is expected to result in a broader phase-separation for immiscible systems. In the above case, the thin-film takes a longer time to organize, then a slow kinetic results in a thermodynamically favoured large crystallization of PCBM domains [127].

**Figure 13:**
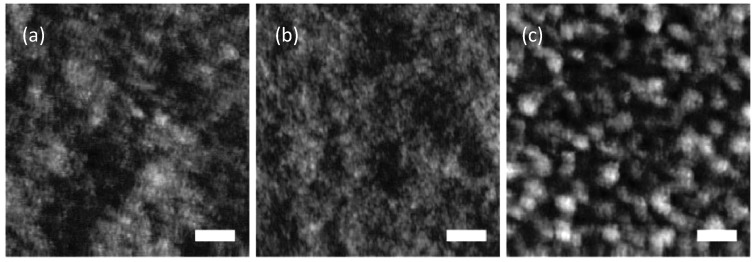
AFM height images of PF10TBT:PCBM BHJs spin-casted from chlorobenzene (see Figure 11 for molecular structures) containing (**a**) 20; (**b**) 50; and (**c**) 80 wt% of PCBM. The horizontal scale bar is 200 nm; z-scale is 6 nm. (Reprinted with permission from Reference [133]. Copyright (2008) by American Chemical Society).

**Figure 14:**
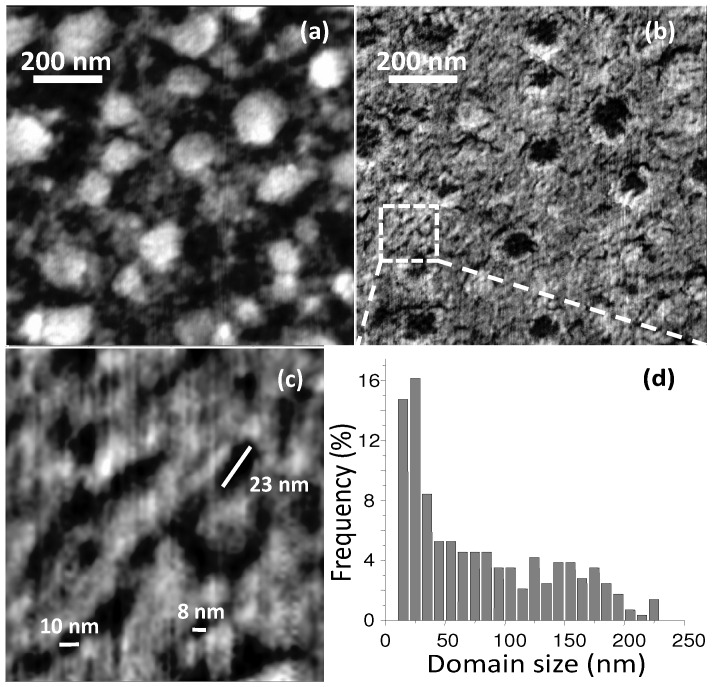
AFM topography (**a**) and phase-lag images; (**b,c**) of 50:50 wt% P3HT:PDB BHJ thin-film; (**d**) Size distribution of the phase-separated domains. (Reprinted with permission from Reference [120]. Copyright (2010) by Wiley-VCH Verlag GmbH & Co.).

Another important parameter that affects the thin film blend morphology is the solution concentration and the ratio of D and A materials. For example in MDMO-PPV:PCBM BHJs, the higher the whole component concentration the larger is the phase separation [130]. As for the D:A ratio, it has been curiously demonstrated for a number of polymer:PCBM blends, that a strong excess of PCBM with a blend ratio of about 1:4 (as for the BHJ in Figure 12) leads to a sudden increase of PCEs along with the expected PCBM phase separation [102,131]. Truly, PCBM gives a poor contribution to the overall amount of light absorbed, however a low content of only 5% is still able to completely quench the D photoluminescence, indicating a quite complete exciton dissociation [132]. Thus, the reason for the enhanced PCEs is that such a high concentration is needed to trigger percolative pathways to the electrode, meaning that in this case, charge transport is the limiting factor more than exciton separation. For example, Figure 13 shows that by increasing the PCBM content from 20% to 80% in a PF10TBT:PCBM BHJ, a nanoscale (>10 nm) phase separation occurs leading to 50–100 nm sized PCBM clusters (Figure 13c) for thin films spin-cast from chlorobenzene. As is visible, at 80 wt% PCBM clusters come together affording a continuous pathway. Indeed, passing from 20 to 50 to 80 wt% of PCBM, the cell PCE increases from about 2% to 2.5% up to 4%, respectively [133]. In another example, P3HT was blended with a Boron-rich polymer as A-material (polyphenylborane; PDB) [120]. By modulating the D:A blending ratio to 80:20 wt%, it was possible to obtain a phase separation of 10–20 nm, which is close to the exciton diffusion length scale of P3HT. Nevertheless, it was demonstrated that the 50:50 wt% blend, although displaying a slightly coarser A:D separation (Figure 14), affords better photovoltaic performances. This is because a trade-off between phase-separation and continuous vertical percolative pathways is needed. Accordingly, the 50:50 wt% BHJ would offer more continuous and effective paths, favouring charges to reach the respective electrodes.

It was demonstrated also that the component molecular weight may affect the morphology. In this regard, van Bavel *et al.* reports an interesting study on the 3D characterization of morphology. These researchers studied the phase separation of a poly(2,7-(9’,9’-dioctyl-fluorene)-alt-5,5-(4’,7’-di-2-thienyl-2’,1’,3’-benzothiadiazole) (PFTBT):PCBM thin-film, reporting for low MW PFTBT a very fine phase separation, whereas for a higher MW an optimal domain size of 10–20 nm comparable to the exciton diffusion length was achieved [134].

**Figure 15:**
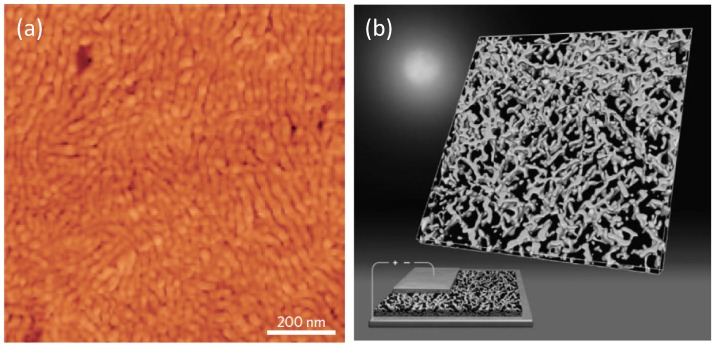
(**a**) AFM image of a solvent-annealed P3HT:PCBM BHJ thin-film. (Reprinted with permission from Reference [60]. Copyright (2007) by Wiley-VCH Verlag GmbH & Co.); (**b**) 3D electron tomography of P3HT nanofibres in a P3HT/PCBM BHJ displaying a bi-continuous percolating network. In the bottom-left corner, the 3D image is integrated in an artistic view on energy conversion by OSCs (Reprinted with permission from Reference [139]. Copyright (2009) by American Chemical Society).

One of the main tools in the three-dimensional control of BHJs is the post-deposition thermal- [135] or solvent-annealing [60,136] or a combination of both. Actually, when a BHJ thin-film undergoes an external stimulus such as solvent vapours or heating above the glass transition temperature (T_g_), molecules gain mobility and diffuse toward a thermodynamically favoured 3D organization. In this way, the post-treatment annealing allows further control of the critical thermodynamic-kinetic interplay enabling an efficient 3D organization in the final BHJ to beobtained. Post-deposition annealing introduces a higher crystallinity by phase separation, partially reducing the D:A total interface useful for exciton separation [137]. However, the increased crystallinity brings an enhanced charge carrier mobility which offsets the loss in the exciton dissociation efficiency, still leading to high PCEs OSCs [138]. As an example, in P3HT:PCBM systems, the post-annealing treatment drives a thermodynamic reorganization of P3HT molecules which form highly crystalline micrometric long nanofibres yielding a conductive and percolative 3D network (see Figure 15). In addition, these fibres act as fences that hinder the diffusion and large phase segregation of PCBM [134,139,140]. The control of PCBM crystallization can be also experienced by using a material with a high glass transition temperature (T_g_) which limits the PCBM diffusion leading to non-equilibrium structures.

The formation of BHJ thin-film is a complex mechanism and non-homogeneous vertical segregation is generally expected [141], especially when the surface free energy of D and A differs significantly. In order to minimize the total free energy, the composition at the substrate/BHJ and at the BHJ/air interfaces should be different depending on the surface free energies of components and of the substrate on which they are deposited [142,143]. As an example, researchers demonstrated such a substrate surface tension dependence for a P3HT:PCBM BHJ. They revealed a vertical concentration gradient with a PCBM-rich region near the substrate/BHJ interface and P3HT-rich domains at the BHJ/air interface [141]. Even in this case, annealing treatment can be used to modulate the vertical diffusion of components and to obtain an effective vertical distribution [142].

Although spin-coating is the most widely used method to obtain high efficiency OSCs, it is unsuitable for large scale deposition since it requires quite rigid substrates of reduced dimension. Nonetheless, in order to access the market, OSC’s technology needs massive deposition tools (such as roll-to-roll deposition [16], ink-jet printing [144], gravure printing [144], *etc*.) which are generally able to guarantee an appropriate control of the morphology.

**Figure 16:**
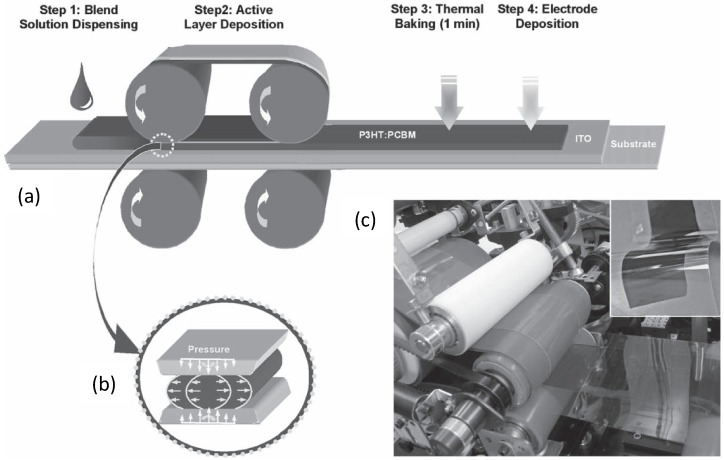
Schematic depiction of a roll-to-roll process for the large scale fabrication of OSCs: (**a**) casting of the D:A blend solution (ITO is indium tin oxide); (**b**) dynamic layer formation by controlled solvent evaporation, the sketch describes the solution shear flow inducing chain alignment; (**c**) A picture of the roll-to-roll apparatus (in the inset, the flexible OSCs before contact deposition). (Reprinted with permission from Reference [145]. Copyright (2010) by Wiley-VCH Verlag GmbH & Co.).

For example, by means of a roll-to-roll based method (see Figure 16), Park *et al.* [145] succeeded in controlling the P3HT:PCBM morphology by placing on the cast film (Figure 16a, step 1) a gas-permeable silicone membrane (Figure 16a, step 2) under pressure for regulating the solvent evaporation rate (Figure 16a, step 2 and b). The pressure induced a shear flow (Figure 16a, step 2 and b) helping to align the D polymer chains while the solvent evaporated through the silicone film. After the removal of the silicon membrane, the BHJ was subjected to a short thermal annealing (1 min) and to the final electrode deposition (Figure 16a, step 3–4). Of course, the thickness of the film obtained is also affected by the solution concentration, roller pressure, and rolling speed [146]. Besides producing OSCs with a rate of 10–20 mm/s, these researchers obtained a finer interpenetrating network along with a more uniform distribution of the blend, higher PCE and charge mobility compared to spin-coated samples.

Gravure printing [147,148] has been also employed to realize OSCs. Voigt *et al.* [147] obtained high quality printed BHJ by treating substrate with plasma and using optimized ink viscosity, substrate surface tension and roughness. Besides, a desirable morphology was achieved by using high boiling point solvents in order to modulate the solvent evaporation rate. These authors prepared Gravure printed OSCs and found that by this method inverted configuration cells had significantly longer lifetime than non-inverted ones.

Moreover, the usage of high boiling point solvents to reduce the drying speed of deposited films is a strategy also exploited in ink-jet printed OSCs [149,150,151]. Eom *et al.* [151] found that the addition of high boiling point additives to the main D:A solution offers great control on the morphology showing improved light absorption, reduced recombination losses due to an improved crystallinity and charge mobility. These authors built ink-jet printed P3HT:PCBM BHJ solar cells having a remarkable PCE of 3.71%.

Finally, a few words must be written on the long-term stability of these devices. Truly, the word “stability” is not appropriate in this case, since we are speaking about far-from-equilibrium structures. Thermodynamically, a structural change of the active film is favoured and it may be even accelerated by the operating conditions (thermal stress by sun irradiation), *i.e.*, an “intrinsic degradation” [152]. Further detrimental processes including the chemical deterioration of electrode metals [153] and polymer molecules in the presence of oxygen and water [154] as well as the photo-oxidation of polymers [155] are grouped under the term “extrinsic degradation” [152]. While extrinsic issues can be fixed by improving the manufacturing technology and by an effective encapsulation [156], intrinsic degradation continues to degrade cells although perfectly sealed and this is one of the main causes of the limited lifetime of OSCs that is the real bottleneck for their successful commercialization.

In a study on the demixing of polythiophene:perylene BHJ, Keivanidis *et al.* [157] observed a decline of the photophysical properties already after two weeks from the layer deposition because of the phase separation of components and the consequent reduction of D:A interface.

Interestingly, it has been proposed that polymer regioregularity may also have a role in the long-term durability of devices [158]. OSCs based on highly regioregular P3HT:PCBM BHJ have shown decreased photoconversion performance upon ageing, because the higher crystallinity of the P3HT favour the segregation of the PCBM reducing the D:A interface. Conversely, low regioregular P3HT:PCBM cells offer lower but more stable conversion efficiency (over four months), since the low regioregular polymer limits the PCBM diffusion. In view of this, the selection of a proper regioregularity grade seems to be a crucial point in order to set a trade-off between high-efficiency and long-durability.

The ease of diffusion is also connected with the mechanical characteristics of polymers which are related to the T_g_. An interesting study on PPV based polymers:PCBM BHJ devices revealed that high T_g_ polymers display an enhanced thermal stability of the BHJ structure with long-lasting performance. This is probably due to the “stiffer” high-T_g_ polymeric matrix that may hinder the diffusion and phase segregation of the PCBM component [159].

## 3. Conclusions

Despite a huge effort over the last three decades, the nanoscopic control of morpology in thin molecular films remains still a matter of basic research. There is no consolidated theoretical framework to predict and explain the non-equilibrium structures of thin films and experimentalists usually work by trial and error procedures to optimize the structures frozen in the confined layers of single polymers or blends over suitable substrates.

However, it is clear that polymers and specifically supra-molecular structures may adapt in 3D in a large variety of complex states and the ability to tailor their functions requires a fine nanoscopic morphological control. Here, we reviewed the morphology-performance relationship by selecting some case studies dealing with a variety of structures achievable also by employing similar systems deposited under different dynamic conditions for devising thin film transistors and OSCs. Explicitly, the increasing massive market in organic and plastic electronics has to take into account the above issues dealing with processing low cost technologies to obtain improved performances together with lowering the cost/performance ratio of devices and systems.
